# Annotations

**Published:** 1890-04-12

**Authors:** 


### Annotations.

**Sausages and Polonies.**

Mr. Lees-Knowles, M.P., contributes an article full of varied instruction to the current number of the *Nineteenth Century*, on the subject of horseflesh. In the course of the article he incidentally mentions certain important facts about the manufacture of cheap sausages and polonies. He states, on the authority of one of the metropolitan medical officers of health, that horseflesh is largely used by low-class sausage makers under the technical trade-name of "Jack." The flesh, after the animal has been killed, is covered with salt in order to absorb external moisture, and to purify the meat from any disagreeable smell or taste. Afterwards it is well soaked in water to get rid of the salt, and then it is ready for the mincing machine. One notorious sausage-maker, who was, it is said, a large buyer of beef before he took to the use of horseflesh, afterwards purchased only the back-fat of pigs to mix with the horseflesh, and bought no more beef. Five or six years ago the medical officer of health for Glasgow discovered that a considerable traffic in horseflesh was being carried on in that city. Quantities of raw horseflesh, invoiced as such, were being sent to Barnsley, whence, after being salted, they were removed to Hull and shipped for Norway. On a particular day in December, 1884, the slaughterer at Barnsley had no less than forty tons of horseflesh on his premises, all of it being salted in preparation for the Norway market. At Stockholm the meat is said to be made into sausages and German polonies. There is no reason whatever why horseflesh should not be used as human food; but it is well for certain classes of persons who have a devoted attachment to sausages, polonies, and other highly-spiced condiments to know exactly what it is they are partaking of with so much relish. The flesh of young and plump horses is quite as wholesome, and may conceivably be as agreeable to the taste as that of cows or pigs. But the personal history of a horse who ends his days at the slaughterer's yard, is such as to render the animal quite unfit for the food of even moderately civilised persons. He is mostly old and lean; often diseased. In many cases the animals are town horses, that have been housed for years in airless stables, and knocked and beaten about until all the vitality has been pounded out of them. Such animals, even when eaten fresh, have hardly any food value; but when kept until decomposition has set in, their flesh cannot but be injurious to the human stomach, and to the blood after digestion. The question for consideration is not that of the edibility of the young and healthy horse, but of the wholesomeness and food value of old, tough-fibred, bloodless, and worn-out creatures that not seldom die a natural death; and even when this is not the case, are apt to be passed from hand to hand so often, that the first stages of putridity have set in before they finally come into the consumer's hands. Cheap sausages and polonies are frequently unwholesome and injurious, and poor people would be well advised to choose by preference the cheap portions of the fresh meat sold by ordinary butchers, and to cook and season them for themselves.

**Mr. Lilly and Mr. Herbert Spencer.**

"See how these Christians love one another," was said in commendation in the early days of the Christian Church. "See how these literary and scientific folks love one another" will probably be the satirical note of the new time. This is Mr. W. S. Lilly's account of Mr. Herbert Spencer: "A thoughtful mind, enriched by patient study of the experimental sciences, so far as they can be studied merely in books—which is not very far" (amiable Mr. Lilly!)—"but apparently unversed in metaphysics and undisciplined by philosophy; self-taught, and unwarrantably disdainful of that long succession of the world's teachers who, from the days of the Vedas to the days of Kant, have done so much to illuminate the dark places of moral and mental science, and to increase the bounds of rational knowledge." This is sufficiently plain speaking. It would be exceedingly interesting to hear Mr. Herbert Spencer's reply, if he could be prevailed upon to say exactly what he thinks of Mr. Lilly after reading his verdict. The interest, for ordinary people, of such an expression of opinion as we have quoted lies in this, that men of distinguished eminence in science and literature set so very little store by each other's judgment and intellectual worth. Whilst nine-tenths of the educated world are bowing the knee to Mr. Spencer, and hailing him as the prophet of the scientific and political future, Mr. Lilly is describing him practically as a mere armchair scientist, who learns his science purely from books, and whose culture is so limited that he neither understands nor even knows what many of the most powerful and original minds in ancient and modern times have thought and taught. One obvious conclusion that ordinary men and women will draw from this is that as the leading minds in science and literature are so little infallible to one another, therefore they should not be held infallible by the world at large. The "plain man," who is not a specialist, is apt to distrust himself. Many, indeed begin to feel that neither in public nor in private should they venture to speak at all, except on the commonest topics of every day experienee. Conversation and the instruction of the people they are told to leave altogether to the specialists. Dreadful conclusion! When the world comes to consist entirely of condensed pedants it will no longer be worth living in. Even now the man who ferrets rats despises him who only kills moles because he does not understand the profound mysteries of the rat-killing specialty. The "plain man" must assert himself in self-defence; and if not in self-defence, then in the interest of his fellows. A world of undifferentiated specialists is too dreadful for the stoutest-hearted to contemplate.

**Was it Hypnotism?**

Mr. Hamilton Aïdé relates a curious experience in the *Nineteenth Century*. It is only a case of table turning, or, rather, of table springing. The table was a polished rosewood piece of furniture of substantial weight, and the operator was Mr. Home, of American fame. Among other witnesses was M. Alphonse Karr, who was as confirmed a sceptic on the whole subject of table turnings and spirit rappings as could well be imagined. In the case related the table rose from three to four feet in the air, and remained at that elevation a considerable time—long enough, indeed, for M. Karr to creep beneath its legs and to search with the most critical minuteness for possible ropes, pulleys, or other "cheating" arrangements. Nothing, however, was found; and to this day Mr. Hamilton Aïdé is still asking himself how it could possibly have happened, and what it could possibly have meant. There must be many other persons who have had similar experiences. The writer remembers with as much distinctness and as much scientific dissatisfaction as Mr. Aïdé the case of a small table which, in the presence, and apparently under the inspiration of a young lady of two-and-twenty, now the wife of a well-known African missionary, took to raising two of its legs in the air, then to oscillating backwards and forwards, and finally to making a very decided aerial movement in the direction of the door. In this, as in Mr. Aïdé's case, there could be no doubt whatever about the facts. The young lady was quite conscious of her own "uncanny" power, and never exercised it except on the most urgent persuasion of her friends. The facts being admitted, we have to ask—What is the explanation of them? At present there is no explanation, and, with true inconsequence, we affect to despise, and even to disbelieve the facts, simply because we cannot explain them. To what a very few people it ever occurs to ask themselves this question—How many of the phenomena of nature, even of those circumstances that are happening every day, do I thoroughly and completely understand? The mysteries of birth and of thought, of gravitation and of force, which were mysteries to Aristotle and Democritus, are as great mysteries to Tyndall and Edison to-day. But it seems such a ridiculous thing for a table to rise up and commence an inconsequent pirouette under the uncertain domination of a man with an abnormal nervous system. It is probable that the inconsequence of the table's movements is due to the want of intensity and energy in the power that produces them. It is, so to speak, rather a beginning, a mere hint of a power than a power in full development. It is quite possible to conceive of a human being with such large powers of this kind, that he might by a mere touch or even a wave of the hand cause all kinds of articles to execute decided and purposive movements, and, in fact, to do his nervous bidding exactly as an intelligent child obeys the spoken word. Why not? There must be power enough of that kind in the space that surrounds us. We are constantly telling each other how little we know, and yet scientific men are always acting as if they ought to have the power of knowing everything, and refuse to have anything at all to do with phenomena which they cannot explain.

